# PARP-1 genetic polymorphism associated with radiation sensitivity of non-small cell lung cancer

**DOI:** 10.3389/pore.2022.1610751

**Published:** 2022-12-14

**Authors:** Hetong Wang, Haitao Xie, Shuying Wang, Jiaying Zhao, Ya Gao, Jun Chen, Yuxia Zhao, Genyan Guo

**Affiliations:** ^1^ Department of Radiation Oncology, The Tenth People’s Hospital of Shenyang, Shenyang, China; ^2^ Department of Radiation Oncology, The Fourth Affiliated Hospital of China Medical University, Shenyang, China; ^3^ Department of Radiation Oncology, Liaoning Cancer Hospital, Shenyang, China; ^4^ Shandong Polytechnic College, Jining, China; ^5^ Department of Radiation Oncology, Qingdao United Family Healthcare, Qingdao, China; ^6^ Department of Oncology, Kailuan Hospital, Tangshan, Hebei, China

**Keywords:** radiotherapy, polymorphism, non-small cell lung cancer (NSCLC), PARP-1, sensitivity

## Abstract

About 70% of non-small cell lung cancer (NSCLC) patients require radiotherapy. However, due to the difference in radiation sensitivity, the treatment outcome may differ for the same pathology and choice of treatment. Poly (ADP-ribose) polymerase 1 (PARP-1) is a key gene responsible for DNA repair and is involved in base excision repair as well as repair of single strand break induced by ionizing radiation and oxidative damage. In order to investigate the relationship between PARP-1 gene polymorphism and radiation sensitivity in NSCLC, we collected 141 primary NSCLC patients undergoing three-dimensional conformal radiotherapy. For each case, the gross tumor volumes (GTV) before radiation and that after 40 Gy radiation were measured to calculate the tumor regression rate. TaqMan real-time polymerase chain reaction was performed to genotype the single-nucleotide polymorphisms (SNPs). Genotype frequencies for PARP-1 genotypes were 14.2% for C/C, 44.7% for C/G and 41.1% for G/G. The average tumor regression rate after 40 Gy radiation therapy was 35.1% ± 0.192. Tumor regression rate of mid-term RT of C/C genotype was 44.6% ± 0.170, which was higher than that of genotype C/G and G/G (32.4% ± 0.196 and 34.8% ± 0.188, respectively) with statistical significance (F = 3.169 *p* = 0.045). The higher tumor regression rate in patients with C/C genotype suggested that G allele was a protective factor against radiation therapy. Using the median tumor regression rate of 34%, we divided the entire cohort into two groups, and found that the frequency distribution of PARP-1 gene rs3219073 had significant difference between these two groups (*p* < 0.05). These results showed that PARP-1 gene polymorphism may affect patient radiation sensitivity and predict the efficacy of radiotherapy. It therefore presents an opportunity for developing new therapeutic targets to improve radiotherapy outcome.

## Introduction

Lung cancer has the highest incidence rate and mortality rate among different malignant tumors [1]. It has a 5-year overall survival rate of less than 15% in USA, and even lower in China [2]. The non-small-cell lung cancer (NSCLC) represents 80%–85% of newly diagnosed lung cancers. Within NSCLC patient population, about 75% patients are un-operable advanced lung cancers. Currently, radiation therapy (RT) is a crucial component for treating these patients. About 64.3% of the patients need RT at different phases during the course of treatment[3, 4]. However, the post-RT tumor regression rate and local control rate can be significantly different among patients. This is mainly due to the difference in radiation sensitivity for each patient, which is closely related to a series of factors such as cell cycle, cell hypoxia, proliferative activity, DNA damage repairing, as well as apoptosis of cells [5–9]. Hence, gene polymorphism, gene mutation and epigenetic modification affecting the underlying radiobiological response can also lead to difference in sensitivity, as interrelated genes have different biological responses to radiation.

DNA repairing mechanism within a cancer cell involves more than 150 genes and five main pathways: base excision repair (BER), nucleotide excision repair (NER), mismatch repair (MMR), double strand break repair (DSBR) and homologous recombination repair (HRR). Each pathway is responsible for repairing different types of DNA damages. The repairing of single strand breaks is mainly performed through BER pathway, which involves key genes such as PARP-1, OGG1, APE1, XRCC1. The BER pathway repairs DNA base damages caused by oxidative reagents and alkylating agents, which plays an important role in the maintenance of DNA integrity [10–12]. It is believed that the ability of BER pathway in repairing damaged DNAs is associated with the radiosensitivity of lung cancer cells.

Poly (ADP-ribose) polymerase 1 (PARP-1) is a key DNA repairing gene closely involved in the BER pathway, and is responsible for single strand break repairing induced by ionizing radiation and oxidative damage [13]. Currently, several types of PARP-1 inhibitors have been studied in clinical trials, which have shown that inhibiting the activity of PARP-1 could inhibit DNA repair and therefore improve the damaging effect of radiotherapy and chemotherapy[14, 15]. In our previous study, it was shown that PARP-1 rs3219073 gene polymorphism was closely associated with the occurrence of lung cancer [16]. In this work, we investigate the effect of PARP-1 gene polymorphism on the radiosensitivity of patient with lung cancer, as well as that on the effectiveness of RT. It is hypothesized that the gene repairing capacity can be affected by PARP-1 gene polymorphism, which might affect the efficacy of RT.

## Materials and methods

### Study population

All the subjects in this work were Han population in the northern region of China without blood ties. This study has been approved by the Ethics Committee of the Fourth Affiliated Hospital of China Medical University (EC-2019-HY-016). With written consent, the peripheral blood samples were collected, and an epidemiological study was then conducted. This study included 141 primary NSCLC patients recruited from our institution between September 2009 and December 2012. All cases were pathologically confirmed and without lung operational resection. Within the patient cohort, 89 were squamous cell carcinoma, and 52 were adenocarcinoma.

### Treatment

All the patient cohort underwent three-dimensional conformal radiotherapy (3D-CRT) treatment consisting of 20 treatment fractions with a total of 40 Gy radiation dose. Within the patient cohort, 88 patients received chemotherapy for 2–4 weeks, and were then treated with radiotherapy 2 weeks after the last chemotherapy and after the hemogram returned normal, the others chose radiotherapy directly and actively without chemotherapy. There was no interruption or chemotherapy during the course of radiotherapy. All patients were simulated using computer tomography (Siemens) in supine, immobilized position. The gross tumor volume (GTV) included the primary tumor and the metastasis lymph node within 1 cm radius or that directly fused with the primary tumor. For each case, the tumor volume was measured before and after treatment. The clinical target volume (CTV) and the planning target volume (PTV) were defined by expanding the GTV with a 6–8 mm margin, and by expanding the CTV with a 6–10 mm margin, respectively. Fixed display window width and window level set by Treatment Planning System (TPS) were used for treatment planning (Pulmonary WW: 1600, WL: -300; mediastinal WW: 400, WL: 800).

3D-CRT was performed using a 6 MV linear accelerator (Siemens), with the 95% prescribed dose line covering 95% of PTV. Patients were re-scanned in the same position at the end of the entire 40 Gy treatment. The tumor target was then re-outlined to update the gross tumor volume (renamed as GTVS), clinical target volume (renamed as CTVS) and planning target volume (renamed as PTVS) after treatment. Tumor regression rate was then calculated from GTV (before RT) and GTVS (after 40 Gy RT) using the following expression: R= (GTV before RT - GTVS after 40 Gy RT)/(GTV before RT) × 100%.

### Gene extraction and classification

Two milliliters of peripheral blood samples were collected from each participant and stored in sodium citrate tubes. DNA was then extracted using proteinase K (Merck) digestion and phenol–chloroform (Dingguo Biology)extraction method. Taqman real-time polymerase chain reaction (RT-PCD) was performed (SDS software, Applied Biosystems) to genotype the single-nucleotide polymorphisms (SNPs). To test the PCD pollution in each 96-well plate and to ensure the accuracy of the genotype results, standard testing procedure using duplicate samples, negative controls without template DNA and double distilled water were performed. 10% of the samples were randomly selected to repeat blind assays. RT-PCR reactions were run in 5 μl mixture including 2.5 μl of Taqman Master Mix (2X), 0.25 μl of primer + probe (20X), 1.25 μl of H_2_O, and 1.00 μl of genomic DNA. Primers, Taqman probes and Master Mix were designed and provided by Applied Biosystems. PCR conditions included initial denaturing step at 95°C for 10 min followed by 47 cycles at 92°C for 30s and then at 60°C for 60s. Allelic Discrimination program of SDS software (Applied Biosystems) was used to detect the fluorescence intensity of FAM and VIC markers by different alleles, as well as to assay genotypes.

### Statistical analysis

Student t tests were used (SPSS 13.0 software) to evaluate the relationship between tumor regression rate and gender, age, and histological type, respectively. Variance analysis was used to evaluate the relationship between the tumor regression rate and the stage. If *p* < 0.05, SNK multiple comparison tests were used, as well as the SNPs. χ2 tests were used to compare genotype distribution between those general characteristics.

## Results

### Tumor regression rate and general characteristics of rs3219073 genotype

The distributions of selected characteristics of 141 lung cancer cases are shown in Table 1. Genotype frequencies for PARP-1 rs3219073 SNP genotypes were 14.2% for C/C, 44.7% for C/G and 41.1% for G/G. No statistically significant correlation was observed between genotypes and gender, age, smoking status, histological type, or clinical stage (*p* > 0.05), as shown in the Table 1 (last column).

**TABLE 1:** Tumor regression rate and general characteristics of rs3219073 genotype.

		Patients	Tumor regression rate of mid radiation therapy (mean ± SD)	*p*-value^a^	Genotype			*p*-value^b^
					C/C	C/G	G/G	
Gender	Male	105	35.0% ± 19.6%	0.886	13	44	48	0.153
	Female	36	35.5% ± 18.2%		7	19	10	
Age	>60	69	36.3% ± 18.3%	0.479	9	32	28	0.895
	≤60	72	34.0% ± 20.1%		11	31	30	
Smoking status	Yes	98	37.3% ± 18.1%	0.047	12	48	38	0.270
	No	43	30.3% ± 20.8%		8	15	20	
Histology	Squamous carcinoma	89	36.8% ± 19.4%	0.192	12	38	39	0.698
	Adenocarcinoma	52	32.4% ± 18.6%		8	25	19	
Pathological stage	I	11	42.6% ± 19.8%	0.507^c^	2	6	3	0.454
	II	20	32.1% ± 18.6%		1	9	10	
	III	84	34.5% ± 19.7%		13	33	38	
	IV	26	36.3% ± 17.7%		4	15	7	
GTV group	Small volume	71	33.0% ± 19.7%	0.177	11	34	26	0.564
	Large volume	70	37.3% ± 18.5%		9	29	32	

^a^ *p*-value was calculated by t test.

^b^ *p*-value was calculated by χ2 test.

^c^ *p*-value was calculated by variance analysis.

The average tumor regression rate after 40 Gy RT was 35.1% ± 19.2%. Three of them presented larger tumor volume than that before treatment (they were −61.1 cm^3^, −3.3 cm^3^, −2.92 cm^3^ respectively). Tumor regression rates of smoking patients were higher than that of non-smoking patients’ (*p* = 0.047). There was no statistically correlation observed between tumor regression rate and gender, age, histological type, or clinical stage (*p* > 0.05).

Although larger volume tumors (volume >= 89.97 cm^3^, medium GTV) shrank more than small volume tumors (<89.97 cm^3^), there was no statistical difference in tumor regression rate between them (*p* = 0.177, Table 1, last row).

### Genetic polymorphisms and curative effect of lung cancer radiotherapy

**FIGURE 1:**
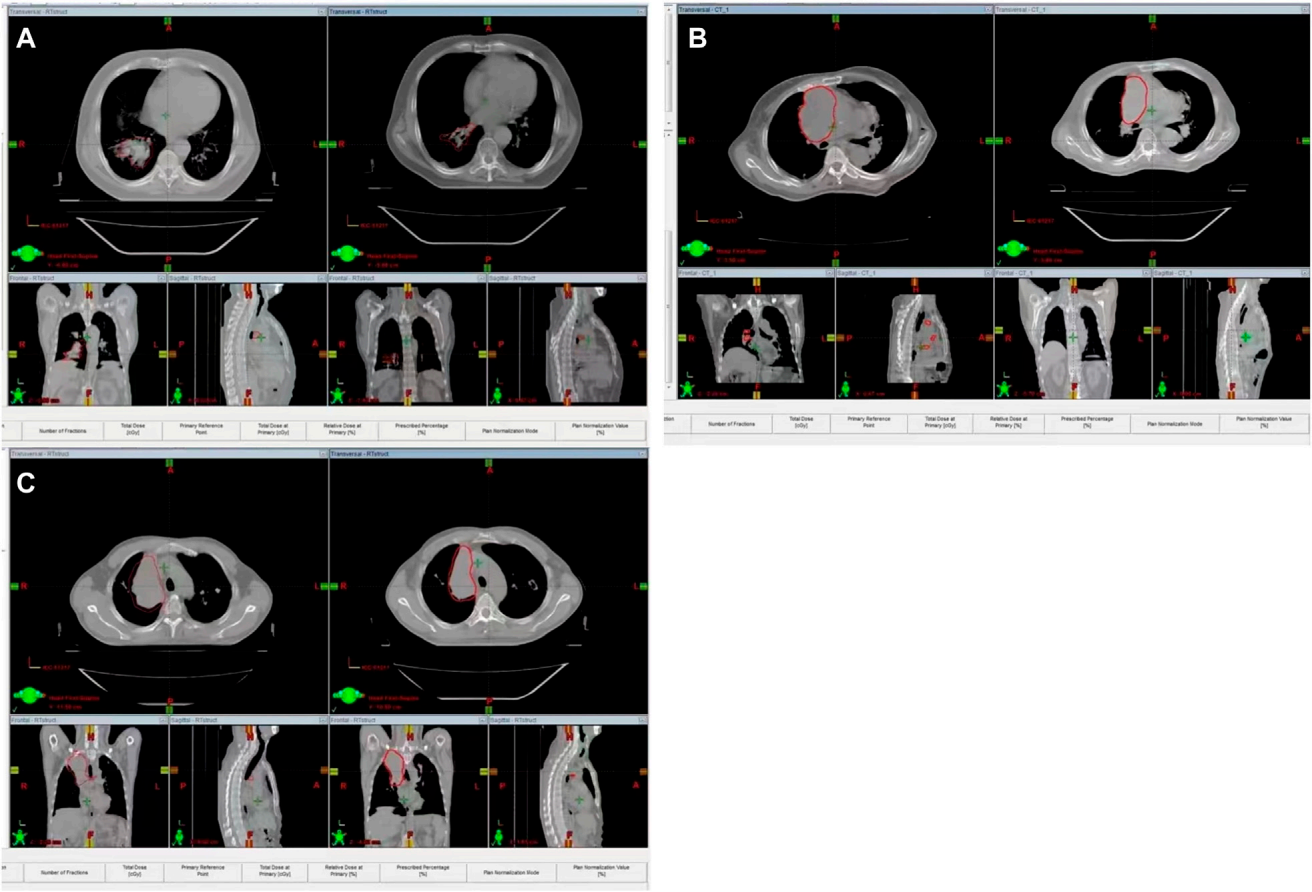
Representative samples of lung cancers with different genotypes before and after radiotherapy. **(A)**: representative samples of lung cancers with CC genotype; **(B)** representative samples of lung cancers with CG genotype; **(C)** representative samples of lung cancers with GG genotype.

The effect of radiotherapy differs for different genotype carriers, as shown in Figure 1. Tumor regression rate of mid-term RT of C/C genotype was 44.6% ± 0.170, which was significantly higher than that of the other two genotypes (C/G + G/G, modified value, 33%) with statistical differences (F = 5.87, *p* = 0.017), as shown in Table 2. In addition, there was significant difference in tumor regression rate between C/C genotype and either of the other two genotypes (C/C vs. C/G, *p* = 0.013; C/C vs. G/G, *p* = 0.048). No significant difference was observed between C/G vs. G/G (*p* = 0.485).

**TABLE 2:** Relationship between gene polymorphism and tumor regression rate of mid radiotherapy.

Genotype	Patients	Tumor regression rate of mid radiation therapy. (mean ± SD)	F-value	*p*-value	LSD *p*-value
C/C	20	44.6% ± 17.0%			—
C/G	63	32.4% ± 19.6%^a^	3.169	0.045	0.013
G/G	58	34.8% ± 18.8%^a^			0.048
C/G + G/G	121	33.6% ± 19.2%^a^			0.017^b^

^a^ Compared with group C/C, *p*-value was calculated by variance analysis.

^b^ *p*-value was calculated by student t test.

We further divided the entire patient cohort into two groups using the median tumor regression rate of 34.0%, and found that the frequency distribution of PARP-1 gene rs3219073 between the two groups had significant difference (*p* < 0.05) (Table 3).

**TABLE 3:** Distribution difference of Gene polymorphisms groups by median value of tumor regression rate.

Genotype	Patients	≤34%	>34%	χ^2^ -value	*p*-value
C/C	20	6 (30.0%)	14 (70.0%)		
C/G	63	38 (60.3%)	25 (39.7%)	6.152	0.045
G/G	58	27 (46.6%)	31 (53.4%)		
C/G + G/G	121	65 (53.7%)	56 (46.3%)	3.863	0.049

*p*-value was calculated by variance analysis.

## Discussion

Studies have demonstrated that radiation therapy can effectively reduce the primary tumor volume, improve local control rate, and may affect the survival time. For example, Fox [17] found that, in the mid-term of RT, the tumor regression rate at 30Gy and 50Gy were 24.7% and 44.3%. Ramsey [18] observed GTV changed every week during the course of RT and found significant decrease of GTV. Although the curative effect of RT is affirmative, tumor shrinkages during RT had been shown to significantly differ among NSCLC patients. Kupelian [19] demonstrated that the tumors with larger original volume had higher tumor regression rate than that with smaller volume. We also had similar observation, despite that no statistical difference was found. Siker [20] and Woodford [21] found that there was no obvious relationship between tumor regression and chemotherapy, pathology, tumor original volume, radiation treatment time, or stages. In this work, we observed that volume changes of primary tumors were not correlated with gender, age, or properties of tumors. Consequently, the possibility that the patient’s own gene mutation and epigenetic modifications result in the differences of radiation sensitivity and tumor regression rate should be considered.

In this study, we found a correlation between primary tumor volume change and smoking status (*p* = 0.047). Studies have shown that cigarette combustion can produce a large amount of oxidation substances and Reactive Oxygen Species (ROS), which might damage cells’ genome, biomembrane, macromolecule, as well as DNA structure [10–13,22,23]. The biological effect of ionizing radiation is mainly explained by the DNA damage due to direct and indirect interaction with the DNA structure of tumor cells. We observed that DNA oxidative damage caused by smoking might enhance the sensitivity of radiotherapy and contributed to a larger tumor volume reduction in this study.

PARP-1, existing in eukaryotic cells, is the nuclear enzyme that catalyzes poly ADP-ribosylation. PARP-1 can selectively recognize and bind with DNA polymerase in DNA gaps to maintain the integrity of the genome and repair the single strand breaks (SSBs). In addition, PARP-1 can participate in DNA double strand breaks (DSBs) repair along the HR and NHEJ pathway in the DNA replication fork [24]. PARP-1 can also recruit protein MRE11, ATM (ataxiatel angiectasia mutated) to suppress the transcription factors E2F4 and P130 complexes and impact the expression of breast cancer susceptibility genes BRCA1 and RAD5 [25], which transiently protects the DNA gaps and inhibits recombinant. PARP1 could interact with XPA, which enhances the activity of PARP1, the DNA damage-binding protein 2 (DDB2), and transcription factor II H (TFIIH), potentially involved in NER pathway. DNA injuries caused by oxidizing agents, alkylating agents and ionizing radiation can rapidly activate PARP-1 [15, 26], and complete the DNA damage repair through the mechanisms mentioned above. Therefore, PARP-l plays an important role in maintaining and repairing genomic integrity.

Inhibition of PARP-l may lead to increased susceptibility to tumor by predisposing the body to DNA damage factor caused by ionizing radiation, and increase the therapeutic effect of chemotherapy and radiotherapy on cancer by decreasing DNA repair function [27, 28]. Chalmers AJ et al. observed the radio-sensitization effect of PARP inhibitors *in vitro* and animal models with lung cancer, colorectal cancer, head and neck cancer, glioma, cervical cancer, and prostate cancer[29]. Many PARP-1 inhibitors had been tested in clinical studies [30–34]. Studies have shown that PARP1 inhibitors are involved in cell migration induced by inhibition of erythropoietin. The mechanism is related to the down-regulation of c-fos and expression of Egr-1 [35]. It was also found that mutation of BRCA1 or BRCA2 was a predictor of PARP inhibitors [33, 34]. These studies indicate that PARP-1 is involved in multiple DNA repair pathways, despite that the mechanism is unclear. In previous studies, we found that the patients carrying G allele had a reduced risk of lung cancer, especially adenocarcinoma. In this study, we excluded the effects of chemotherapy and operation on the curative effects of radiotherapy, and evaluated the radiosensitivity of NSCLC patients using tumor regression rate at mid-RT of 40 Gy/20f. We found that rs3219073 gene polymorphism may affect the radiation sensitivity. Patients with C/C genotype had higher tumor regression rate than that with C/G and G/G genotype (*p* = 0.045). C/C genotype had significant difference from C/G and G/G genotypes in terms of tumor regression rate (*p* = 0.013, *p* = 0.048), respectively. Carrying the G allele is related with reduced tumor regression rate (*p* = 0.017), which suggests that the G allele is a protective factor against RT. On the other hand, patients carrying C allele may be more sensitive to radiation therapy. As far as we know, this is the first study to report the relationship between PARP-1 genetic polymorphisms and radiation sensitivity of NSCLC. DNA is the major target of cell injury caused by ionizing radiation through SSB and DSB. Although part of them could be quickly and fidelity repaired by repair genes, it is the main mechanism of tumor cells killing by ionizing radiation [29, 36]. The rs3219073 C allele gene may suppress the activity of PARP-1 repair gene, which increases lethal cellular damage, inhibits damage repair, and consequently increases the sensitivity and efficacy of RT [37]. Of course, further clinical trials and infra-tests are required to confirm this theory. In summary, PARP-1 gene rs3219073 polymorphism may predict the efficacy of radiotherapy and provide new therapeutic targets to improve the radiotherapy sensitivity of NSCLC patients.

## Data Availability

The data presented in the study are included in the article. Further inquiries can be directed to the corresponding author.
